# Women with learning disabilities and access to cervical screening: retrospective cohort study using case control methods

**DOI:** 10.1186/1471-2458-8-30

**Published:** 2008-01-24

**Authors:** Fiona Reynolds, Debbi Stanistreet, Peter Elton

**Affiliations:** 1 Clinical Epidemiology and Public Health Unit, University of Manchester, Manchester, UK; 2 Department of Public Health, University of Liverpool, Liverpool, UK; 3 Bury PCT, Bury, UK

## Abstract

**Background:**

Several studies in the UK have suggested that women with learning disabilities may be less likely to receive cervical screening tests and a previous local study in had found that GPs considered screening unnecessary for women with learning disabilities. This study set out to ascertain whether women with learning disabilities are more likely to be ceased from a cervical screening programme than women without; and to examine the reasons given for ceasing women with learning disabilities. It was carried out in Bury, Heywood-and-Middleton and Rochdale.

**Methods:**

Carried out using retrospective cohort study methods, women with learning disabilities were identified by Read code; and their cervical screening records were compared with the Call-and-Recall records of women without learning disabilities in order to examine their screening histories. Analysis was carried out using case-control methods – 1:2 (women with learning disabilities: women without learning disabilities), calculating odds ratios.

**Results:**

267 women's records were compared with the records of 534 women without learning disabilities. Women with learning disabilities had an odds ratio (OR) of 0.48 (Confidence Interval (CI) 0.38 – 0.58; *X*^2^: 72.227; p.value <.001) of receiving a cervical screening test; an OR of 2.05 (CI 1.88 – 2.22; *X*^2^: 24.236; p.value <.001) of being ceased from screening; and an OR of 0.14 (CI 0.001 – 0.28; *X*^2^: 286.341; p.value <0.001 of being a non-responder compared to age and practice-matched women without learning disabilities.

**Conclusion:**

The reasons given for ceasing and/or not screening suggest that merely being coded as having a learning disability is **not **the sole reason for these actions. There are training needs among smear takers regarding appropriate reasons not to screen and providing screening for women with learning disabilities.

## Background

There are no official statistics for the number of people with learning disabilities in the UK, but the Foundation for People with Learning Disabilities (FPLD) estimates that the number is 580,000–1,750,000 (2004a). The incidence is thought to be stable (NHS Executive, 1999) though prevalence is increasing as people with learning disabilities are living longer (Sperling, 1997). The number of people with severe learning disabilities is expected to increase by 1% every year over the next 15 years; and as survival rates for people with learning disabilities improve, illnesses associated with ageing (e.g. cancer) are likely to become more prominent [1].

In 2005, cervical cancer killed 1,061 women in the UK [2]. Risk factors for cervical cancer include: smoking, use of oral contraceptives, parity, cervical trauma during birth, (Brinton 1992) and sexually transmitted infections (Lehtinen *et al *1996). However it is possible to develop the disease without ever being sexually active (Raffle *et al*, 2003).

Screening is effective in reducing mortality from cervical cancer [3] and, since 1988, deaths from cervical cancer in England and Wales dropped by five per cent each year and, in Scotland, by four per cent each year. Northern Ireland also saw a two per cent drop in deaths per year [4]. Screening is offered to every woman aged 25–64 and the National Health Service Cancer Screening Programme [5] recommends that age (reaching 65) and absence of cervix are the **only **clinical reasons for ceasing a woman.

Ceasing has the effect of stopping all invitations being sent to a woman and removing her permanently from the Prior Notifications Lists (PNLs) which are those used by the Call-and-Recall system to invite women for screening [5]. This means that the woman will cease to be invited for further screening and this Study also intended to find out whether women with learning disabilities were more likely to be ceased than women without. There are two criteria for this taking place – age and absence of cervix (congenital or following a hysterectomy) – though women can request to be ceased, usually by asking not be sent invitations [5].

Screening is rarely offered to women with learning disabilities and coverage is lower than for women in the general population [6]. Brent and Harrow Health Authority found that only 19% of women with learning disabilities had received screening while 77% of the women had no screening records, 7). Pearson *et al*, [8] discovered that 37% of women with learning disabilities were ceased because they had a learning disability. However:

*"Learning disabilities alone are not a reason for ceasing women from the programme...There will be a small number who are unable to consider the concept of participation and who become distressed when the procedure is attempted." *(5)

Although, there is no evidence that cervical screening should not be performed to the same indications and contraindications as for the general population [9], a recent study carried out by Smith [10] in Rochdale suggests that General Practitioners (GPs) considered cervical screening unnecessary for women with learning disabilities.

There is limited information available about the sexual experiences of people with learning disabilities, however it is clear that a proportion of women with learning disabilities will have experienced consenting and/or nonconsenting sexual relationships [11-13].

This paper describes a study carried out in the North West of England to ascertain whether women with learning disabilities are more likely to be ceased than women without; and to examine the reasons given for ceasing women with learning disabilities.

Bury PCT leads the cervical screening programme for Bury, Rochdale and Heywood-and-Middleton PCTs. Several systems are in place to ensure the goal coverage of 80% of the population [14]. These include the Call-and-Recall System which manages the routine recalling of women for screening [15]. The 'operators' communicate with GPs to ensure that information is up-to-date and that women are receiving the care they need.

## Methods

A retrospective cohort study was carried out to compare the uptake of cervical screening and the likelihood of being ceased, between women with and without learning disabilities.

The study population was women aged 25–64 in the Primary care Trust (PCT) areas of Bury, Heywood and Middleton and Rochdale.

Using *Epi.Info Stat.Calc*, we calculated that a sample size of **217 women with learning disabilities and 434 women without **was required assuming prevalence of screening to be 80% and based on an OR of 2, and a sample ratio of 1:2 (women with learning disabilities to women without) Significance was set at 0.05 and power at 80%.

The case group comprised 267 women with learning disabilities who were in contact with the Learning Disabilities Teams or the GPs in the three areas. 'Controls' for each case were the next two women on the Call-and-Recall Database list, before or after, the NHS numbered woman with learning disabilities who were registered with same practice and within +/-5 years of age.

### Ethics Committee

North Manchester LREC 05/Q1406/82

#### Obtaining Consent for Records to be Accessed

This study was carried out by accessing patient records. Although people caring for a woman with learning disabilities (carers), whether family or a paid employee, cannot consent on her behalf, the Mental Capacity Act (2005) advises that they (or nominated third parties) should be consulted to discover whether the person with learning disabilities would assent to joining any research project. A letter was sent to 267 women with learning disabilities requesting their permission to access their records. In the event that the women did not understand the letter, it was anticipated that they would pass it to their carer. In some cases, carers/parents contacted the PCT office to enquire further about the study.

The letter stated that if the women (or their carers) did not wish to give permission they should contact the study. 46 people contacted the office. The carers of four women withheld consent on the grounds that the woman could not consent. Of the 42 people who wished to find out more 37 were parents who stated that their daughters had never had a screening test but that the women assented to being included. Five were women with learning disabilities who wished to know more and gave their consent.

#### Design and Process

This study was undertaken across Bury, Heywood-and-Middleton and Rochdale PCTs. There were 34 GP practices in Bury, 21 in Rochdale and 14 in Heywood-and-Middleton and work with these took place September-December, 2005. Patient data were stored on the database systems *Vision, EMIS, EMIS PC4 *and *Torex*.

The following Read Codes (used for diagnoses and treatment) were used to identify women on GP systems identified as having learning disabilities. The reason for carrying out this second process of ascertainment was to ensure that as complete a list as possible, of women with learning disabilities, was obtained.

Table 1: Read codes for learning disabilities used in this study.

**Table 1:** Read codes for learning disabilities used in this study.

**Read Codes**	**Definitions**
E3	Mental Retardation
E30..	Mild Mental Retardation
E310.	Moderate Mental Retardation
E311.	Severe Mental Retardation
E312.	Profound Mental Retardation
E140.	Autism
Eu842	Rett's Syndrome
N726./PKy93	Prader-Willi Syndrome
N721/PJ0../PJ0z.	Down 's syndrome
N724/PJ2../PJ2z.	Edward's Syndrome
PKyz5	Angelman Syndrome
N725/PJyy4	Fragile X Syndrome
C301.	Phenylketonuria

Cytology Read Codes were used to identify whether any of the women with learning disabilities were eligible for cervical screening; whether any had received a cervical screening test in the last three years or ever and also to identify reasons given for not having a cervical screening test. The criteria for an adequate cervical screening test was that the women's records showed that a test had been completed within the previous five years.

#### Outcomes

The two groups were compared for:

• Frequency of cervical smear in the previous five years

• Non-response to invitation

• Ceased from call and recall

#### Analysis

Analysis was carried out using SPSS 12 to assess whether eligible women with learning disabilities were less likely to have received an adequate cervical screening test than eligible women without. (The criteria for an adequate cervical screening test was that the women's records showed that a test had been completed within the previous five years). Odds ratios, X^2 ^statistics and p.values, with 95% confidence intervals were used to assess whether women with learning disabilities were significantly less likely to have received a cervical screening test than women without. Analysis took into account women who had reached the upper age threshold or had a hysterectomy in the previous five years.

## Results

225 women were identified through learning disabilities teams and 42 further women were identified via the GP databases bringing the total of women with learning disabilities to 267. 534 controls matched for age (within 5 years) and GP practice without learning disabilities were then identified.

Stratification by learning disability was attempted but the results were not statistically significant. In addition, the use of Read Codes varies across the three areas. In Bury and Heywood-and-Middleton the largest code proportion was actually 'unknown' i.e. the Surgeries had not yet completed Read coding the learning disabilities of these women. In Rochdale, this was the second largest share. Rochdale and Heywood-and-Middleton PCTs had written to all GPs to instruct them to code all patients with learning disabilities under the codes E3 (mental retardation) and Eu81z (learning disabilities). These do not tell us whether there are any particular learning disabilities (e.g. Down 's syndrome) which may be common or unusual. These factors meant that any results from stratification would likely be inaccurate.

Looking first at eligibility for screening, the same proportions from the two groups were eligible for screening (93%). However as shown in this study, the situation regarding ceasing and screening is very different. Of the 267 women with learning disabilities, 251 of them were considered to be eligible for screening and seven were exempted through age and nine because of hysterectomy.

The information regarding women without learning disabilities was taken solely from the Call-and-Recall Records, which do not contain as much information as GP records so these provided sparse information about screening histories other than attendance and ceasing for age and hysterectomy. For example, the records show whether a woman has been ceased or not; but if she has been ceased, only two reasons are documented – age and hysterectomy – which means for the women without learning disabilities there are a number of women who were ceased and no reason is indicated. These women could have refused screening or it may have been considered inappropriate to offer screening for another reason such as physical disability or at this point in time because of illness. (Reasons were obtained for women with learning disabilities by cross-referencing with GP records). Of the 534 controls 498 were eligible. 32 were ceased on the grounds of age and four due to hysterectomy.

In 4 cases it was not possible to determine eligibility for screening (three women with learning disabilities and one woman without) – none of them were outside of the recommended age for screening and there was no evidence that the women had been screened.

Table 2: *Numbers and proportions of women, with and without learning disabilities, who had received (or not) screening in the last three years*.

**Table 2:** Numbers and proportions of women, with and without learning disabilities, who had received (or not) screening in the last three years.

	**Received Screening**	**Had not received Screening**
**Women with Learning Disabilities**	n = 72 (27%)	n = 195 (73%)
**Women without Learning Disabilities**	n = 405 (76%)	n = 129 (24%

Result: Odds Ratio: **0.48**

95% Confidence Interval: **0.38–0.58**

Chi^2 ^statistic: **72.227**

Significance: **<0.0005**

Table 2 shows that women with learning disabilities were 0.48 times less likely to have been screened, or approximately half as likely to have received a screening test in the last five years (52%, 95% CI42% to 62%). If we discount the women who have been ceased because of their age or hysterectomy, we find that of the women with learning disabilities 68 had received screening and 183 hadn't. Of the women without learning disabilities, 394 have received screening while 102 hadn't. The odds ratio calculated on the basis of these figures is 0.46 (95% CI 0.36 – 0.56) – not much different.

Some of the women (four with learning disabilities and nine without) who had received screening in the previous five years were also documented as being ceased during the study as they had now reached 64, and so could be exempted through age.

Women with learning disabilities made up 66% of the total number of women who had been ceased. Table 3 shows that the odds of women with learning disabilities being ceased from the cervical screening programme was more than two times higher than women without even after taking into account women who had reached the upper age threshold or had had a hysterectomy in the last five years.

**Table 3:** Numbers and proportions of women, with and without learning disabilities, who were ceased in the last three years; with calculations of odds ratio.

	**Ceased**	**Not ceased**
**Women with Learning Disabilities**	n = 127 (47%)	n = 140 (53%)
**Women without Learning Disabilities**	n = 68 (12%)	n = 466 (88%)

Result: Odds Ratio: **2.05**

95% Confidence Interval: **1.88–2.22**

Chi^2 ^statistic: **24.236**

Significance: **.005**

### Non-response to screening invitation

Tables 4 shows the likelihood of the two groups responding to a screening invitation. Women with learning disabilities were significantly less likely to respond to invitations for screening than women without with an OR of 0.13 (CI 0.098 – 0.181) even after adjusting for those who had already been ceased.

**Table 4:** Numbers and proportions women, with and without learning disabilities who respond (or don't) to invitations to; with calculations of odds ratio.

	**Responder**	**Non-Responder**
**Women with Learning Disabilities**	n = 90 (64%)	N = 50 (36%)
**Women without Learning Disabilities**	n = 371 (79%)	N = 95 (21%)

Result: Odds Ratio: **0.13**

95% Confidence Interval: **0.098–0.181**

Chi^2 ^statistic: **243.230**

Significance: <**.0005**

An interesting point is that in this group, women without learning disabilities, the national cervical screening target of 80% of the population had not been reached which may suggest that further work to improve the uptake of screening with this group is also necessary.

## Discussion

In this study, women with learning disabilities were significantly more likely to be ceased from screening raising concerns about equity of access to screening programmes. However, it should be noted that the spectrum of learning disabilities within the case group was quite wide, and as this was a small study, it could be misleading. If there was more data available in Read Codes (to explain why 'screening not wanted'), it may be possible to identify those who would benefit from screening and should therefore not be excluded; from those for whom screening might be genuinely inappropriate.

For example, it may be possible that the amount of support given to women with learning disabilities could have some impact on whether they utilise screening services. It can be difficult for people with learning disabilities to access care and treatment, especially screening services (NHS Executive, 1999; FPLD, 2004b). Barriers to accessing services include fear of examinations and difficulties in accessing professionals who link to services (Kopac *et al*, 2004). However, more women would accept cervical screening if their doctors recommended it (Heller and Marks, 2002).

The fact that, statistically, women with learning disabilities were significantly more likely to be ceased from the screening programme and less likely to receive screening than women without, is consistent with previous research locally [12] and elsewhere [9] that women with learning disabilities do not receive the same service as women without. However the extent to which this was the case, was less marked than in previous studies [10].

The reasons given for ceasing and/or not screening suggest that merely being coded as having a learning disability is **not **the sole reason for these actions. However, there are usually additional factors to be taken into account – for example, patient refusal.

There was a large proportion of women with learning disabilities who either did not attend the screening appointment or refused the test. Several of the Read codes identify similar, if not the same concepts (e.g. 6853: screening not wanted and 685L: screening refused). Twenty-three women were documented as either: *did not attend; refused *or *did not want screening*. This may suggest problems with non-response because of fear or not understanding. Those documented as being *due for screening *also shows that although efforts were made to invite these women for screening, there was a problem with non-response.

Other reasons for ceasing or not screening a woman included physical disabilities, which prevented a woman getting herself into the required position. In another two cases attempts were made to carry out a screening test but the cervix could not be reached.

However, for women who had not received screening there wasn't always a reason given. There were 73 women whose records noted that no screening history was documented. This is of concern because: firstly, there is an issue over whether these women are receiving equitable access to services; and secondly, the GP practices are required to keep patient records up to date. One reason for not being able to find a screening history could be a problem with the way the search was carried out – searching for specific Read codes that are not used may have resulted in some records not being shown.

### Comparison with women without learning disabilities

The data concerning the screening situation of women without learning disabilities was taken from the call-and-recall records. The strength in doing this was that it enabled the Study to obtain appropriate controls from an impartial party. However, as stated

Therefore this study can only draw conclusions based on figures for screening, ceasing and non-response to invitations for screening.

The difference between the two groups may be a difference in communication skills and understanding: women without learning disabilities may be more likely to seek advice if they are concerned; or smear takers may find it easier to explain to them, the purpose and process of screening. It may be useful to establish whether sexual inactivity was considered an appropriate reason for not screening in women without learning disabilities.

### Implications

The fact that these women have an increased likelihood of being ceased or not receiving screening, (than women without) indicates that PCTs still have improvements to make, spell out here in more detail what PCT's could do e.g. assessing screening venues to make the process less intimidating, ensuring that LD teams are aware of all patients with learning disabilities so that they can offer support. It may also be necessary to examine invitation processes – e.g. developing tailored letters or processes by which the LD Teams could also be notified that a screening invitation has been sent out.

The lack of Read coding was an issue that was addressed as a result of this study. Once a practice was visited the LD Teams were notified if there were gaps in coding so that they could assist. The lack of coding shows that the practices have not been able to meet the deadline of coding by 2004, which may have a 'knock-on' effect on further work dependent on this being completed.

It is likely that the findings are comparable to the experiences of other PCTs. Some areas may have better or worse screening coverage.

### "How this fits in"

• Like other studies, this research found that women with learning disabilities are less likely to receive a cervical screening test than women without disabilities.

• The findings also support the view that women with learning disabilities are more likely to be ceased from screening than women without, i.e. not receive invitations for screening.

• However, it also found that none of the women with learning disabilities had been ceased *solely *because of their disability.

## Conclusion

The reasons given for ceasing and/or not screening suggest that merely being coded as having a learning disability is **not **the sole reason for these actions. There was a need to improve coding, though this matter was resolved during the process of the study. There does seem to be a need to improve communication between LD Teams and GPs so that patients and staff can be better supported.

## Competing interests

The author(s) declare that they have no competing interests.

## Authors' contributions

FR carried out the study and wrote this report. DS and PE supervised the study, providing support and guidance. All authors read and approved the final manuscript.

## Pre-publication history

The pre-publication history for this paper can be accessed here:


